# The Impact of Post-COVID-19 Condition on Frontline Healthcare Workers: A Scoping Review

**DOI:** 10.1155/cjid/1790795

**Published:** 2025-07-22

**Authors:** Elsie Duff, Em Pijl, Cindy Fehr, Sai Krishna Gudi

**Affiliations:** ^1^College of Nursing, University of Manitoba, Winnipeg, Manitoba, Canada; ^2^Nurse Practitioner Association of Manitoba, Winnipeg, Manitoba, Canada

**Keywords:** COVID-19, healthcare workers, healthcare workforce, pandemic, physical and mental wellness, post-COVID-19 conditions

## Abstract

The main goal of this integrative scoping review was to address the knowledge gap and inform policy and research regarding the impact of post-COVID-19 conditions on frontline healthcare workers (HCWs). An integrative scoping review using Arksey and O'Malley's framework examined post-COVID-19 conditions in frontline HCWs. We searched CINAHL, EMBASE, APA PsycINFO, PubMed, Social Science Database, ProQuest, Social Science Journals, and Web of Science, including dissertations, conference proceedings, and government publications for gray literature. A preestablished data extraction tool was developed to capture relevant information about post-COVID-19 conditions in HCWs. Of the total 42 studies, the majority were cross-sectional in design (29) and conducted mainly in countries such as Italy (4), India (3), and Brazil (3). Study findings reveal that a substantial proportion of HCWs in various countries were diagnosed with post-COVID-19 condition, which included persistent symptoms affecting physical and mental well-being. Persistent symptoms, particularly fatigue and anxiety, were associated with a poorer quality of life, decreased work ability, and impaired health-related quality of life among HCWs. Fatigue was a frequently reported symptom in many studies, often accompanied by weakness, muscle pain, shortness of breath, anxiety, depression, and sleep disturbances. The evidence generated through this research examining post-COVID-19 conditions among HCWs is a foundation for informing policy in the healthcare workforce. These findings also address the gap in research on the broader impacts of the COVID-19 pandemic on employers and the healthcare workforce.

## 1. Introduction

During the coronavirus disease 2019 (COVID-19) pandemic, healthcare workers (HCWs) faced multiple physical and psychological challenges while carrying out their duties [1]. Frontline HCWs were particularly vulnerable to respiratory droplets and aerosol transmission, owing to their work to contain the disease [2]. In addition to the risk of becoming infected with COVID-19, HCWs also suffered from insomnia, loneliness, sleep disorders, and mental depression due to the workload demands and related stress [3]. HCWs also experienced anxiety and frustration due to uncertainty, lack of knowledge, environmental changes, and fear of being infected and carrying the infection to their respective family members [3]. That being said, HCWs' concerns related to maintaining physical distance from their family members to reduce the risk of infection resulted in further psychological distress [4]. Thus, exceptional attention to monitoring the psychological issues of HCWs exposed to COVID-19 becomes essential [5], where research is needed.

COVID-19 compounded the mental health of HCWs due to the high number of cases and deaths of patients and coworkers, not to mention the fear of being infected and carrying the virus to their homes [6]. Other factors of concern included precarious working conditions, long shifts, and lack of personal protective equipment (PPE) [7]. Anxiety has been a common mental health–related symptom presented by HCWs during the COVID-19 pandemic [8]. However, the nature and extent of the impact of the COVID-19 pandemic on HCWs' mental health are largely unknown.

The World Health Organization (WHO) defines post-COVID condition (PCC) symptoms as the persistence or relapse of symptoms at least 12 weeks after the onset of COVID-19 without an alternative diagnosis [9]. The symptoms reported in a recent meta-analysis concluded that HCWs encountered a range of enduring psychological stressors, including anxiety (37%), sadness (36%), and insomnia (32%) [2]. While PCC includes an extensive array of symptoms or difficulties apparent in everyday living, neurocognitive symptoms such as fatigue, memory or concentration problems, and sleep disruption are among the most frequent and debilitating [10, 11]. Large-scale epidemiological studies quantifying the memory, concentration, and executive dysfunction among HCWs with PCC are still lacking. In particular, the impact of PCC on cognitive dysfunction needs to consider the potential contributions of fatigue and psychological distress, which are known to have deleterious effects on cognitive abilities [12]. Fatigue and psychological distress are highly prevalent among HCWs in the context of the additional workload caused by the pandemic, where these symptoms are largely unexplored in research. Therefore, it is important to examine PCC in HCWs to understand the broader impact of the COVID-19 pandemic on the healthcare workforce, inform policy, and identify vulnerabilities that impact HCWs.

## 2. Methods

### 2.1. Study Design

An integrative scoping review was conducted to identify essential PCC concepts important to support the health and well-being of HCWs. The Arksey and O'Malley [13] six-stage scoping review framework was used to integrate various sources, including guidelines, editorials, and gray literature (which is a helpful strategy, especially when there is limited evidence on the topic of interest). The framework was utilized to locate, summarize, and integrate the best evidence regarding the extent and nature of the impact of PCC among HCWs.

### 2.2. Search Strategy

The initial search terms were determined using research questions to build a comprehensive search strategy: *What effect has PCC (*i.e.*, physical or psychological symptoms 12 weeks post COVID-19 infection,* e.g.*, fatigue, memory problems, sleep disturbances, shortness of breath, anxiety and depression, general pain and discomfort, difficulty thinking or concentrating, and posttraumatic stress disorder) had on HCWs?* Relevant keywords such as “long-COVID,” “post-COVID-19,” “post-COVID condition,” “PCC,” “PCC symptoms,” “healthcare workers,” “health personnel,” and “frontline healthcare workers” were searched with the subject headings from each database.

For instance, the detailed search strategy interpretation using PubMed was as follows: (“Post-Acute COVID-19 Syndrome”[MeSH Terms] OR “long haul covid”[Title/Abstract] OR “post covid”[All Fields] OR “Post acute covid”[Title/Abstract] OR “long COVID”[Title/Abstract] OR “PCC”[Title/Abstract] OR “pcc symptom^∗^”[Title/Abstract]) AND (“Health Personnel”[MeSH Terms] OR “frontline”[Title/Abstract] OR “healthcare personnel”[Title/Abstract] OR “health care personnel”[Title/Abstract] OR “healthcare worker^∗^”[Title/Abstract] OR “health care worker^∗^”[Title/Abstract] OR “health care provider^∗^”[Title/Abstract] OR “healthcare provider^∗^”[Title/Abstract] OR “Health Care Professional”[Title/Abstract] OR “healthcare professional^∗^”[Title/Abstract] OR “health care professional^∗^”[Title/Abstract] OR “hospital worker^∗^”[Title/Abstract] OR “hospital personnel”[Title/Abstract] OR “medical worker^∗^”[Title/Abstract] OR “medical provider^∗^”[Title/Abstract] OR “hospital employee”[Title/Abstract] OR “healthcare employee^∗^”[Title/Abstract] OR “hospital staff^∗^”[Title/Abstract] OR “healthcare staff”[Title/Abstract] OR “health care staff”[Title/Abstract]). The search strategy was reviewed by a research librarian at the authors' university. Preliminary criteria considered gray literature and English-language research studies from January 01, 2020, through April 07, 2025.

### 2.3. Study Selection

**Figure 1:**
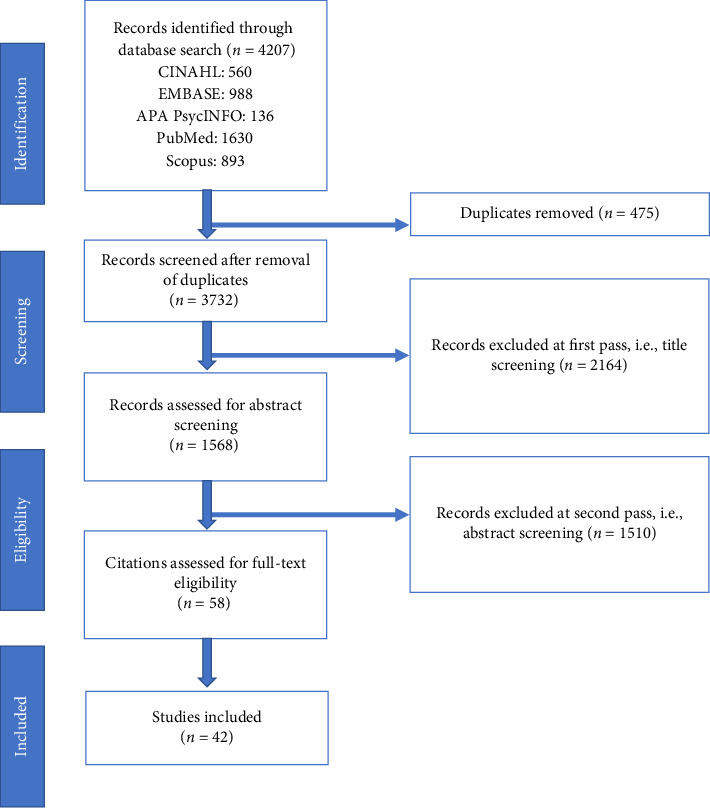
PRISMA flowchart illustrating the study selection process.

Electronic scientific databases such as CINAHL with full-text EBSCOhost, EMBASE-Ovid, APA PsycINFO-Ovid, PubMed, and Scopus databases were searched, including gray literature through hand searching. Initial research produced 4207 records, where 475 duplicate studies were removed using Zotero. At the second pass, i.e., title screening, 2164 studies were excluded, whereas at the third pass, i.e., abstract screening, an additional 1510 studies were excluded. After thoroughly reviewing full-text articles, 16 studies were excluded (Figure 1). A research coordinator searched, retrieved, and completed the data extraction of articles using the Zotero reference management system. In collaboration with the research coordinator, two independent investigators from the research team reviewed the abstracts, coded the articles, and decided if the inclusion criteria had been met. Ultimately, 42 studies were included in this scoping review. Articles relevant to PCC among HCWs with symptoms of physical and mental wellness (i.e., fatigue, memory problems, sleep disturbances, shortness of breath, anxiety and depression, general pain and discomfort, difficulty thinking or concentrating, or stress) were included. The characteristics and primary findings of the included studies are presented in Table 1.

The research team developed a preestablished data extraction tool to capture participant demographics, professional designation, sample size, measures/assessments, outcomes, results, and author conclusions relevant to PCC among HCWs. Once the independent reviews were completed, the investigators compared the results and discussed any differences, returning to the original study for the rationale to support their opinion. The third reviewer decided to include or exclude the study based on the criteria identified in the data extraction and review process if the team could not reach a consensus.

### 2.4. Data Charting

Zotero reference management software [56] was used to store the articles, note comments on inclusion and exclusion, and summarize the extracted data. The Zotero software was also used to identify articles to an “INCLUDE” folder and to remove duplicate citations. Summaries included identifying the reviewer, research question(s), study purpose, country of origin, study methodology, participants, sample size, measurements used, theories identified, results, limitations, relevance to the research questions, reviewer's recommendation, and a synopsis of the article.

## 3. Results

Of the 42 studies included in the review, the majority were cross-sectional studies (*n* = 29), followed by prospective/retrospective (*n* = 6), case-control (*n* = 3), multimethod/descriptive (*n* = 2), and mixed methods (*n* = 2) studies. Geographically, most of the included studies were conducted in Italy (*n* = 4), India (*n* = 3), Brazil (*n* = 3), Saudi Arabia (*n* = 2), United Kingdom (*n* = 2), China (*n* = 2), Egypt (*n* = 2), England (*n* = 2), Germany (*n* = 2), Portugal (*n* = 2), Switzerland (*n* = 2), Turkey (*n* = 2), and the USA (*n* = 2), followed by one study each in Canada, Indonesia, France, Latin America, Ireland, Jordan, Palestine, Philippines, South Africa, Sweden, Malaysia, and Tunisia. These investigations collectively aimed to analyze various aspects of HCWs' PCC experiences and outcomes.

## 4. Discussion

This scoping review serves as a fundamental step in informing healthcare workforce policy to address the broader impacts of the COVID-19 pandemic. Across multiple countries and study designs, HCWs who had contracted COVID-19 commonly experienced a range of persistent symptoms. Below is the list of reported PCC symptoms from the included literature based on the severity and frequency of occurrence.

### 4.1. Symptom Implications

#### 4.1.1. Fatigue

Fatigue was the most common symptom reported by HCWs [14–32, 34–55, 57], which often co-occurred with other symptoms. All the included studies in this review indicated a significant prevalence of severe fatigue among HCWs. In Germany, 11% of HCWs reported experiencing severe fatigue, correlating with heightened psychological distress, diminished quality of life, and increased work incapacity [58]. Similarly, a study conducted in China found that 76% of HCWs with severe COVID-19 continued to experience symptoms of fatigue and weakness even 28 months post-discharge, with 18.7% failing to recover their functionality fully [23]. The persistence of symptoms, particularly fatigue and anxiety, has been linked to a decline in overall quality of life, decreased work ability, and impaired health-related quality of life among HCWs [24]. Notably, fatigue emerged as the most frequently reported symptom among HCWs in Italy, affecting 32.1% of individuals studied, followed by musculoskeletal pain (13.6%) and dyspnea (13.2%) [24]. Furthermore, research from Turkey highlighted the long-term impact of COVID-19 on young patients, with mild and moderate cases experiencing PCC symptoms that significantly impacted their quality of life [21]. Among German HCWs, 31.5% reported persistent impairment in their quality of life, underscoring the persistent effects of COVID-19 on HCWs [31]. A common occurrence of concentration, brain fog, or memory deficiency was reported with long COVID-19 symptoms in HCWs [15, 21, 22, 27, 29–32, 34, 37–40, 51, 52, 57]. These findings emphasize the need for targeted interventions and support mechanisms to address the complex challenges posed by post-COVID fatigue and its ramifications on individuals' well-being and occupational functioning.

#### 4.1.2. Muscle or Joint Pain

Frontline HCWs who recovered from COVID-19 frequently reported persistent musculoskeletal symptoms, including muscle and joint pain [39, 40, 42, 44–48, 51–53]. Studies consistently indicate that these symptoms can significantly increase in intensity and frequency post-infection [25], whereas the most frequent symptom in the post-acute phase includes muscle aches. Furthermore, a significant proportion of post-COVID-19 patients met the criteria for fibromyalgia, with prevalence rates ranging from 31% to 40% [17]. In a similar study, HCWs reported the most common presenting symptoms as fatigue (73.6%) and muscle or joint pain (73.6%) [20]. The long-term impact of COVID-19 on musculoskeletal health among HCWs is particularly concerning, given the physically demanding nature of their roles. Persistent pain and reduced physical activity can significantly decline the quality of life and work capacity. Therefore, it is crucial to implement comprehensive rehabilitation programs and pain management protocols tailored to the needs of the essential workforce [23, 24]. Muscle or joint pain is vital for HCWs' safety, ergonomics, wellness, or disability management to address, preventing injury or loss of function for work productivity by employers.

#### 4.1.3. Headache

Following fatigue and joint or muscle pain, headache was reported to be one of the most frequent symptoms among HCWs [39, 44–48, 51, 54]. In Jordan, almost three-quarters (71.4%) of HCWs who participated in the study reported headache as one of the most frequent symptoms of COVID-19 infection during the acute and follow-up phases of infection [20]. The prevalence of headache as a post-COVID symptom among HCWs in Italy was found to be more than 27% [25]. Similarly, a recent study conducted in the USA identified headache as a frequently reported symptom, even after 6 weeks of COVID-19 infection, posing a substantial disease burden among HCWs [28]. A similar German study reported that more than 70% and 41% of the HCWs experienced headaches during COVID-19 infection and post-COVID-19 syndrome, respectively [30]. Lastly, a recent study in India reported that around 35% and 15% of the HCWs experienced headaches during active illness and post-COVID-19 recovery, respectively [32]. Headache is a common reason for work absences, impacting essential HCW human resources and patient care.

#### 4.1.4. Depression

Compared to the general public, the overall depression scores among recovered HCWs were reported to be higher (mean DASS-21 score of 25.9) [15]. A notable percentage of HCWs still experience normal to mild depression, especially HCWs under 30 years of age, who exhibit higher depression levels than their older counterparts. This study also found that longer quarantine durations were associated with increased depression levels, underscoring the psychological toll of prolonged isolation. Wang et al. [37] provided evidence that preexisting mental health conditions and preinfection psychological distress, including depression, anxiety, perceived stress, loneliness, and worry about COVID-19, significantly increased the risk of developing PCC symptoms and associated daily life impairments. In a study by Gaber et al. [18], 44% of respondents reported mood disorders, including depression, during the post-COVID-19 period. Despite acknowledging the severity of their symptoms, many HCWs were reluctant to seek medical advice or take sick leave, potentially exacerbating chronic symptoms due to maladaptive coping strategies [18]. Additionally, Mendola et al. [27] noted persistent anxiety and depression among HCWs even 10 months after recovery, significantly impacting their workability and fitness to work. That is, the post-COVID syndrome is linked with reduced work ability, with recommendations that modifications in workload and shifts to aid recovery are needed [25]. These findings collectively underscore the critical need for ongoing mental health support and monitoring for HCWs recovering from COVID-19. Addressing the psychological impacts, particularly depression, through targeted interventions and supportive workplace policies is essential to mitigate the long-term consequences of PCC symptoms in this vital workforce [39–41, 45].

#### 4.1.5. Anxiety and Stress

The unprecedented circumstances and associated unwanted consequences of the COVID-19 pandemic triggered significant stress among both the public and frontline HCWs [39, 41, 43, 45]. Recent studies reported severe posttraumatic stress symptoms following PCC, especially among HCWs who are young [15, 36, 37]. Younger HCWs, especially those with underlying conditions like asthma, were more likely to experience anxiety, with anxiety and depression being closely linked [37]. Carascal et al. [15] found that recovered HCWs experienced higher levels of distress and anxiety compared to the general population. Factors such as age, tenure, and length of quarantine significantly influenced anxiety levels. Magnavita et al. [25] identified that workers experiencing symptoms for more than 4 weeks exhibited higher levels of anxiety, depression, and fatigue and showed reduced work ability. This group often saw a resurgence of preexisting conditions, leading to a notable decline in their overall quality of life and work performance. The long-term persistence of anxiety and depression post-COVID-19 was noted by Mendola et al. [27] who found that while physical symptoms decreased 10 months after recovery, anxiety and depression remained prevalent. Tempany et al. [34] corroborated these findings, with 22% of HCWs in their study reporting residual psychological symptoms, primarily anxiety, after recovering from COVID-19. These findings are significant as the mental health challenges among post-COVID-19 HCWs are a burden for the workforce.

#### 4.1.6. Sleep Disturbance

HCWs recovering from COVID-19 reported significant cognitive impairments, including difficulties with concentration [43, 48–50], memory, and experiences of “brain fog.” These cognitive issues persisted long term and impacted HCWs' ability to perform tasks efficiently [21]. Moreover, studies consistently highlighted cognitive symptoms as part of the broader spectrum of post-COVID-19 conditions, emphasizing the need for ongoing support and interventions for affected individuals [15, 27, 29, 30, 32, 57]. Several studies emphasized the impact of sleep disturbances among HCWs recovering from COVID-19 [19, 25, 27, 34, 37], significantly affecting physical and mental health, workability, and overall well-being. These studies accentuate the need for interventions to improve sleep quality to support HCWs' recovery and enhance workforce sustainability [19, 25, 27, 34].

#### 4.1.7. Chest Pain

Besides psychological symptoms, PCC also affects the cardiovascular system, where chest pain is frequently reported [17, 38, 39, 45, 57] with palpitations [17] among HCWs with PCC. In the Czech Republic, around 8% of the HCWs reported experiencing chest pain or pressure during the post-acute COVID-19 syndrome [57]. Furthermore, in a South Africa study, 24% of frontline HCWs reported experiencing chest pain during the post-acute COVID-19 syndrome [38]. A similar study in Egypt reported that around 23% and 9% of HCWs experienced chest pain during acute and acute post-COVID-19 infection, respectively [17]. Such symptoms would impact health human resources as chest pain requires a physical assessment and can result in work absence.

#### 4.1.8. Dyspnea

A significant prevalence of dyspnea among HCWs experiencing post-COVID-19 syndrome is evident in the literature [40, 41, 44–47, 52, 53]. D'Ávila et al. [16] identified that 36.2% of 174 HCWs were diagnosed with post-COVID-19 syndrome, with dyspnea affecting 12.7% of this group. This finding is consistent with Fouad et al. [17] who noted that dyspnea was the most frequently reported symptom among 140 HCWs with chronic post-acute COVID-19. Gaber et al. [18] reported that 45% of 138 HCWs experienced persistent symptoms, with dyspnea, anxiety, and sleep disturbances being nearly universal among those still symptomatic 3–4 months after the peak of the COVID-19 wave. Kaplan et al. [21] further highlighted that dyspnea, along with fatigue, was prevalent among HCWs diagnosed with COVID-19, particularly for those whose symptoms persisted over 12 weeks. Respondents also indicated shortness of breath as a persistent symptom post-COVID, with Peters et al. [30] reporting this symptom among 73% of surveyed HCWs and Platten et al. [31] reporting 30.1%. Magnavita et al. [25] observed that dyspnea was present in 20% of their study cohort, along with other persistent symptoms such as fatigue and sensory disturbances. These symptoms, including dyspnea, were comparable to those experienced by patients with severe forms of COVID-19. However, the frequency of dyspnea was lower in cases of mild infection compared to hospitalized patients. Breathlessness was a common health issue in HCWs post-COVID-19 [32]. Moreover, Mendola et al. [27] found that 18 months post-infection, exertional dyspnea and dyspnea at rest remained significant symptoms among HCWs, with 71% reporting at least one COVID-like symptom and 76% experiencing ongoing symptoms 6 weeks after illness onset. Mohr et al. [28] observed that dyspnea was among the symptoms most strongly associated with having no prior vaccination, with 76% of participants reporting persistent symptoms 6 weeks after onset. For employers, dyspnea in the HCW workforce would be essential to consider in relation to the loss of function for work productivity.

#### 4.1.9. Loss of Smell and Taste

Loss of smell and loss of taste during COVID-19 infection is believed to be likely due to damage to the olfactory bulb by the SARS-CoV-2 virus and is one of the most commonly reported symptoms at 12 months post-infection [22]. A study conducted in Turkey to investigate the post-COVID-19 syndrome in HCWs revealed that almost half of the study participants experienced loss of smell (49.6%) and loss of taste (47.1%) during the diagnosis of COVID-19 infection [21]. Around a quarter of the participants still exhibit loss of smell (22.3%) and loss of taste (19%) even after 3 weeks of the infection. Furthermore, loss of smell (13.2% and 11.6%) and loss of taste (9.1% and 6.4%) were persistent among participants after 12 and 24 weeks of COVID-19 infection, respectively [21, 26, 57]. Interestingly, loss of smell was reported more frequently in the acute and post-infection phases than loss of taste. Another study from India noted that the loss of smell and taste (21%) persists beyond recovery from active illness [32]. A survey that examined HCWs' consequences of COVID-19 infection, the risk factors, and the impact on quality of life over time in Germany concluded that loss of smell and taste were severe symptoms in both acute (63.5%) and post-COVID-19 (38.1%) phases [30]. Living with loss of smell or taste can affect one's quality of life beyond enjoying food or healthy nutrition, and for the HCW workforce, the sense of smell is critical to health assessment or identifying safe workplace environments [40, 44, 48].

#### 4.1.10. Sex or Gender Implications

Considering the aspects of sex or gender, several studies observed that female HCWs often experienced PCC symptoms for a longer period compared to males [14, 20, 22]. These findings might be due to the absence of necessary resources for women HCWs. Other study findings reported menstrual changes [20, 57] and higher scores for females having persistent PCC symptoms [15, 18, 19, 25, 35, 41, 42]. Female HCWs worldwide are facing the downstream effects of their work, including mental health issues, increased physical violence, alternative arrangements for their families not to expose them to risk, and physical exhaustion [58]. These adverse differences between sex and gender in the context of PCC research are critical to examine in health workforce studies to develop policy or to support HCWs. Female HCWs who experience PCC require support, rehabilitation, and possibly financial compensation [31].

#### 4.1.11. Older Age HCWs

Recent evidence suggests that older age is a potential risk factor for developing PCC symptoms [42]. However, long-term symptoms of COVID-19 among older adults are highly debated. A retrospective cohort study of HCWs infected with COVID-19 inferred that older age was associated with symptoms persisting over 3 and 12 months. However, in most cases, those reported symptoms were considered mild [26]. In an Italian study, older HCWs were more likely to experience PCC symptoms, especially musculoskeletal pain, dyspnea, and fatigue [24]. However, there is mixed evidence regarding older age and its association with long COVID-19 symptoms. For example, meta-analyses did not support a higher risk of long COVID-19 symptoms associated with older age (age as a continuous variable and categorizing older adults as 60 years and above) [59]. A recent cohort study that assessed psychological and physical recovery among Chinese HCWs with severe COVID-19 at 28 months after discharge concluded that older HCWs with severe COVID-19 recovered slowly compared to their younger counterparts in terms of health-related quality of life, persistent symptoms, functional fitness, and immune function at 28 months after discharge [23]. Different definitions of long-term COVID-19 infection or PCC used in various analyses might explain the differences in these inconsistent findings. Therefore, the significance of old age as a risk factor for developing PCC symptoms requires further investigation.

## 5. Limitations

This scoping review included articles written and published only in English. The articles examined HCWs from various countries but cannot be generalized concerning PCC and HCWs.

## 6. Conclusion

This integrative literature review aimed to identify the broader impacts of the COVID-19 pandemic on HCWs and healthcare employers that may be utilized to inform policy or identify vulnerabilities that impact the workforce. Beyond knowledge creation, these findings are essential for employers of frontline healthcare clinicians to understand the profound effects on the health workforce's physical and mental health, including their quality of life. The findings highlight the considerable vulnerabilities within the healthcare sector that compel unified efforts to address the enduring PCC health issues among HCWs.

## Figures and Tables

**Table 1:** Characteristics of included studies aimed at the HCWs' PCC experiences and outcomes.

Author, year, and country	Study design	Study population and sample	Key findings
Abukhalil et al., 2022 [14], Palestine	Cross-sectional survey	HCWs *n* = 389	Fatigue, weakness, and muscle pain were the most reported symptoms. Female HCWs experienced longer symptom durations compared to males.

Carascal et al., 2022 [15], Philippines	Mixed-method study	HCWs *n* = 93	Recovered HCWs showed high COVID-19-related distress, mild depression, significant anxiety levels, and stress influenced by sociodemographic and quarantine factors.

D'Ávila et al., 2023 [16], Brazil	Prospective cohort study	HCWs *n* = 641	36.2% of HCWs had PCC, leading to a significant deterioration in health-related quality of life.

Fouad et al., 2022 [17], Egypt	Case-control study	HCWs *n* = 69	PCC resulted in higher fatigue levels and functional limitation but did not significantly impact work performance.

Gaber et al., 2021 [18], England	Cross-sectional survey	HCWs *n* = 3579	Moderate-to-severe fatigue was the most disabling symptom (39%), and other symptoms like shortness of breath, anxiety, and sleep disturbance were common in subjects with persistent symptoms.

Grazzini et al., 2022 [19], Italy	Cross-sectional survey study	HCWs *n* = 427	Over a third of HCWs reported residual symptoms 3 months after SARS-CoV-2 infection, with fatigue and sleep disorders being prominent.

Hyassat et al., 2023 [20], Jordan	Cross-sectional survey	HCWs *n* = 140	59.3% of HCWs reported PCC, with fatigue being the most frequent symptom.

Kaplan et al., 2022 [21], Turkey	Cross-sectional survey	HCWs *n* = 121	Young patients with mild and moderate COVID-19 infection experienced long-term PCC affecting their quality of life.

Kisiel et al., 2022 [22], Sweden	Cross-sectional survey	HCWs *n* = 366	47% reported persistent symptoms 12 months after COVID-19 diagnosis.

Li et al., 2023 [23], China	Cross-sectional longitudinal survey	HCWs *n* = 271	76% of HCWs with severe COVID-19 had symptoms of fatigue/weakness at 28 months post-discharge; 18.7% did not fully recover functional fitness.

Lulli et al., 2023 [24], Italy	Cross-sectional survey	HCWs *n* = 318	Fatigue (32.1%) was the most reported symptom, followed by musculoskeletal pain (13.6%) and dyspnea (13.2%).

Magnavita et al., 2023 [25], Italy	Cross-sectional survey	HCWs *n* = 1378	HCWs with PCC had poorer quality sleep, increased fatigue, anxiety, depression, and decreased work ability compared to those with rapidly disappearing symptoms.

Martinez et al., 2021 [26], Switzerland	Cross-sectional survey	HCWs *n* = 7637	26.5% and 13.5% reported persistent symptoms at 3 and 12 months, respectively, with fatigue, impaired taste/smell, and weakness being common.

Mendola et al., 2022 [27], Italy	Cross-sectional survey	HCWs *n* = 56	Physical well-being symptoms decreased, but memory and anxiety/depression persisted 10 months after recovery.

Mohr et al., 2023 [28], USA	Prospective survey	HCWs *n* = 419	COVID-19 mRNA vaccine reduced symptom prevalence and hastened return to work for HCWs with COVID-19 illness.

Omar et al., 2022 [29], Egypt	Cross-sectional survey	HCWs *n* = 92	Post-infection with COVID-19 correlated with anxiety and depression scores and inversely correlated with attention and memory.

Peters et al., 2022 [30], Germany	Cross-sectional survey	HCWs *n* = 2053	Participants with persistent symptoms had an impaired health-related quality of life, highlighting a need for rehabilitation.

Platten et al., 2022 [31], Germany	Multi-method (descriptive and clinical data)	HCWs *n* = 1506	31.5% reported continued impairment in quality of life among those with persistent symptoms.

Rao et al., 2021 [32], India	Cross-sectional survey	HCWs *n* = 163	Post-recovery, 66% experienced health issues, with fatigue and mild exertion being the most common symptoms.

Stephens et al., 2023 [33], England	Cross-sectional clinical antibodies	HCWs *n* = 305	Fatigue (47.5%), shortness of breath, muscle/joint aches, loss of smell, headache, and sleep disorders were typical symptoms of PCC.

Tempany et al., 2021 [34], Ireland	Cross-sectional survey	HCWs *n* = 144	71% reported persistent symptoms, with 41% being asymptomatic since the pandemic's start. Fatigue (4%).

Ts et al., 2023 [35], India	Cross-sectional survey	HCWs *n* = 201	Despite COVID-19 vaccination, sleep and quality of life remained impaired among younger HCWs.

Uvais et al., 2022 [36], India	Cross-sectional survey	HCWs *n* = 107	The prevalence of depression, anxiety, and PTSD among PCC patients was 26.2%, 12.1%, and 3.7%, respectively.

Wang et al., 2022 [37], USA	Prospective cohort survey	HCWs *n* = 54,960	Depression, anxiety, stress, loneliness, and worry about COVID-19 were associated with a higher risk of self-reported PCC over 1 year.

Wose Kinge et al., 2022 [38], South Africa	Cross-sectional survey	*n* = 207	Common persistent symptoms include fatigue, anxiety, difficulty sleeping, chest pain, muscle pain, and brain fog.

AlBahrani et al., 2023 [39], Saudi Arabia	Cross-sectional survey	HCWs *n* = 243	Cough, shortness of breath, muscle ache, headache, sore throat, diarrhea, and loss of taste were the most common symptoms at the start of the COVID-19 illness. Hair loss, cough, and diarrhea were the main symptoms that lasted > 3 months.

AlBahrani et al., 2025 [40], Saudi Arabia	Cross-sectional survey	HCWs *n* = 80	Fatigue, wheezes, myalgia, and palpitations were the most common post-COVID-19 symptoms. Study reveals that COVID-19 infection did not substantially impair the pulmonary functional capacity of HCWs.

Azeredo et al., 2024 [41], Brazil	Retrospective cohort study	HCWs *n* = 463	Almost half of the HCWs experienced post-COVID-19 syndrome. Fatigue, memory disorders, dyspnea, anxiety/depression, and cough were the most persistent COVID-19 symptoms.

Chen et al., 2024 [42], China	Cross-sectional survey	HCWs *n* = 4757	The prevalence of long COVID-19 among HCWs was 12.6%. Hypomnesia, sleep difficulties, fatigue, disturbances in the reproductive system, hair loss, and myalgia/arthralgia were the most common long COVID-19 symptoms.

Dempsey et al., 2024 [43], United Kingdom	Longitudinal survey	HCWs *n* = 5248	One-third of the HCWs reported prolonged COVID-19 symptoms, while more than 7% reported a post-COVID-19 syndrome. Fatigue, difficulty in concentrating, insomnia, and anxiety or depression were the most common PCC symptoms.

Fki et al., 2025 [44], Tunisia	Cross-sectional survey	HCWs *n* = 249	Almost two-thirds of the HCWs reported post-COVID-19 syndrome. Breathlessness, fatigue, memory impairment, chest pain, and joint pain were the most persistent symptoms.

Lim et al., 2024 [45], Malaysia	Cross-sectional survey	HCWs *n* = 609	Fatigue, cough, decreased physical strength, and musculoskeletal pain were the most common symptoms of post-acute COVID-19 syndrome. This study also highlights that those who received booster vaccinations were less likely to develop PCC symptoms.

Marra et al., 2023 [46], Brazil	Case-control study	HCWs *n* = 7051	Headache, myalgia/arthralgia, nasal congestion, fatigue, fever, dyspnea, cough, and sore throat were the most frequent long-term COVID-19 symptoms. Those infected with the SARS-CoV-2 delta or omicron variant, and those receiving 4 COVID-19 vaccination doses before infection, were less likely to develop long-term COVID-19.

Marques et al., 2023 [47], Portugal	Case-control study	HCWs *n* = 326	Headache and cognitive complaints were the most prevalent neurological complaints reported by the HCWs in the study.

Nehme et al., 2023 [48], Switzerland	Prospective longitudinal cohort study	HCWs *n* = 900	Fatigue, headache, insomnia, cognitive impairment, stress/burnout, pain, digestive symptoms, dyspnea, and cough were the most commonly reported symptoms.

Ozkan et al., 2024 [49], Turkey	Descriptive study	HCWs *n* = 166	Difficulty performing daily activities, fatigue, forgetfulness, and weakness were the most commonly reported PCC symptoms.

Prazeres et al., 2025 [50], Portugal	Cross-sectional survey	HCWs *n* = 348	Extreme fatigue, cognitive dysfunction, shortness of breath, and persistent cough were the most commonly reported long-COVID-19 symptoms.

Saade et al., 2024 [51], France	Cross-sectional survey	HCWs *n* = 1062	One-tenth of HCWs reported post-COVID. Fatigue, loss of taste/smell, dyspnea, cough, and cognitive impairment were the most reported symptoms upon return to work.

Sinaga et al., 2024 [52], Indonesia	Cross-sectional survey	HCWs *n* = 100	23% of HCWs experienced mild to moderate fatigue, while only 1% had reported severe fatigue. Significant associations were found between PCC symptoms (confusion, insomnia, myalgia, arthralgia, throat pain, headache, and chest pain) and fatigue.

Tajer et al., 2024 [53], Latin America	Cross-sectional survey	HCWs *n* = 2030	Dyspnea, fatigue, chest pain, palpitations, cough, anosmia, insomnia, headache, impaired concentration, slowness, impaired memory, depression, anxiety, nausea, and dizziness were the most frequently reported long COVID-19 symptoms.

Torrance et al., 2024 [54], United Kingdom	Mixed-methods study	HCWs *n* = 471	Fatigue, brain fog, breathlessness, sleep disturbance, and heart palpitations were the most prevalent long COVID-19 symptoms.

Zadunayski et al., 2025 [55], Canada	Prospective cohort study	HCWs *n* = 995	The most commonly reported classic post-COVID symptoms were fatigue and shortness of breath, followed by cognitive dysfunction, pain, mental ill-health symptoms, and sleep problems. Other common nonclassic symptoms reported were cough, headache, and altered taste or smell.

## Data Availability

No new data were generated.
